# Vivisection

**Published:** 1920-09-25

**Authors:** 


### "VIVISECTION."

THE latest Home Office Return shows that during 1919 the number of persons holding licences entitling them to perform experiments upon animals was 714, and of these 352 performed none at all. Otherwise the total number of experiments performed with anæsthetics was 4,459, and without anæsthetics 58,228, the total being thus 14,733 fewer than in the previous year. The nature of this work is perhaps made more apparent to the uninitiated when it is pointed out that over 24,000 of these experiments were performed by public health authorities in the course of their necessary work, while over 25,C00 were employed in connection with the essential testing of antitoxic sera, vaccines, and drugs.

The regulations, which are, indeed, strictly enforced, make ample provision for the prevention of all unnecessary suffering, and it is well that the public should know these facts. "Vivisection" as now practised is not a dark and shrouded mystery. In no case has a person received a certificate dispensing with the use of anæsthetics for operations more severe than the opening of a superficial vein. After any of this work, if pain is likely to continue, or where serious injury has been inflicted on the animal, it is always required that death must be caused before the animal regains consciousness. Also, strictly antiseptic operations only are allowed, and if suppuration occurs later the animal must always be killed. In the ordinary run of this work, as will be seen from the figures quoted above, the great majority of the tests made are those requiring no anæsthetic, and it is also to be remembered that these are mostly quite simple procedures as compared with what is popularly known as a "surgical operation." Mostly they are inoculations, feeding experiments, or the administration of various substances by the mouth or by inhalation, or else abstraction of blood by simple or vene-puncture.

And, above all, when consulting the statistics, let it be noted that in a very great number of these straightforward tests the results are negative—and the animals suffer little or no inconvenience beyond the initial handling by the operator, which is never in the least drastic. They have to be kept under observation in ordinary comfortable cages and runs for a short time. To the majority nothing at all happens, and those which show signs of illness are promptly and painlessly killed and submitted to a *post-mortem* examination. To be allowed to perform even the simplest of these experiments every operator must be highly experienced in such work, he must hold the requisite Home Office certificates, and official inspections are both frequent and thorough.

